# Enhancing Electrocardiogram Classification with Multiple Datasets and Distant Transfer Learning

**DOI:** 10.3390/bioengineering9110683

**Published:** 2022-11-11

**Authors:** Kwok Tai Chui, Brij B. Gupta, Mingbo Zhao, Areej Malibari, Varsha Arya, Wadee Alhalabi, Miguel Torres Ruiz

**Affiliations:** 1 Department of Electronic Engineering and Computer Science, School of Science and Technology, Hong Kong Metropolitan University, Hong Kong, China; 2 International Center for AI and Cyber Security Research and Innovations, Department of Computer Science and Information Engineering, Asia University, Taichung 41354, Taiwan; 3 Lebanese American University, Beirut 1102, Lebanon; 4 Center for Interdisciplinary Research, University of Petroleum and Energy Studies (UPES), Dehradun 248007, India; 5 Department of Computer Science, King Abdulaziz University, Jeddah 21589, Saudi Arabia; 6 School of Information Science & Technology, Donghua University, Shanghai 200051, China; 7 Department of Industrial and Systems Engineering, College of Engineering, Princess Nourah Bint Abdulrahman University, Riyadh 11671, Saudi Arabia; 8 Insights2Techinfo, India; 9 Immersive Virtual Reality Research Group, Department of Computer Science, King Abdulaziz University, Jeddah 21589, Saudi Arabia; 10 Department of Computer Science, Dar Alhekma University, Jeddah 22246, Saudi Arabia; 11 Instituto Politécnico Nacional, CIC, UPALM-Zacatenco, Mexico City 07320, Mexico

**Keywords:** auxiliary domain, cardiovascular disease, deep learning, distant transfer learning, electrocardiogram (ECG), heterogeneous datasets, knowledge transfer, negative transfer

## Abstract

Electrocardiogram classification is crucial for various applications such as the medical diagnosis of cardiovascular diseases, the level of heart damage, and stress. One of the typical challenges of electrocardiogram classification problems is the small size of the datasets, which may lead to limitation in the performance of the classification models, particularly for models based on deep-learning algorithms. Transfer learning has demonstrated effectiveness in transferring knowledge from a source model with a similar domain and can enhance the performance of the target model. Nevertheless, the consideration of datasets with similar domains restricts the selection of source domains. In this paper, electrocardiogram classification was enhanced by distant transfer learning where a generative-adversarial-network-based auxiliary domain with a domain-feature-classifier negative-transfer-avoidance (GANAD-DFCNTA) algorithm was proposed to bridge the knowledge transfer from distant sources to target domains. To evaluate the performance of the proposed algorithm, eight benchmark datasets were chosen, with four from electrocardiogram datasets and four from the following distant domains: ImageNet, COCO, WordNet, and Sentiment140. The results showed an average accuracy improvement of 3.67 to 4.89%. The proposed algorithm was also compared with existing works using traditional transfer learning, revealing an average accuracy improvement of 0.303–5.19%. Ablation studies confirmed the effectiveness of the components of GANAD-DFCNTA.

## 1. Introduction

Electrocardiograms (ECG) have been helping human beings in medical monitoring and diagnosis for more than a century. Machine learning algorithms were applied to formulate ECG classification problems such as the detection of various types of cardiovascular disease [1], heart muscle damage detection [2], stress detection [3], and drowsiness detection [4]. Large-scale medical data collection is challenging due to privacy [5], ethics [6], and security [7]. This usually results in small-scale electrocardiogram dataset collection by medical institutions and research groups. In the algorithmic perspective, deep-learning algorithms have superior advantages in the enhancement of models when sufficient training data is available [8,9]. There were some studies suggesting the applicability of deep-support vector machines for small-scale datasets [10].

Attention is drawn to transfer learning, which could leverage the performance of the target model based on the pretrained source model. Typically, both the source and target datasets have similar domains and modalities. It is observed in related works that transfer learning can improve the performance of the target model to a certain extent; however, there is room to achieve excellent performance. In regard to ECG classification, where the problem is often encountered, biased classification and small-scale datasets occur. The vision to apply the deep-learning algorithm to enhance the performance of the classification model is required to overcome potential model overfitting with limited training data. In view of this concern, the extension of the problem formulation of transfer learning with heterogenous datasets between source and target domains was considered in this article. This is an emergent research area, namely distant transfer learning, which has benefits such as the unlimited possibilities in choosing pretrained source models, particularly for source domains that are highly dissimilar to the target domain. The pretrained source models are trained with large-scale datasets that could benefit the fine-tuning of the target ECG classification models in new knowledge, the reduction of model overfitting and biased classification, etc. Nevertheless, distant transfer learning experiences a tradeoff in the key challenges in the design of new auxiliary domains and negative transfer avoidance algorithms. With the success of distant transfer learning, the enhancement of the performance of the target model can be achieved by both similar and distant source domains.

In the following, a literature review was conducted to study the methodology and results of the existing works. Several research limitations were observed, which served as the rationale of our proposal: to address the limitations of the existing works.

### 1.1. Literature Review

To the best of our findings, there were no related works in distant transfer learning using auxiliary domains for ECG classifications. Therefore, the discussion in this subsection considered traditional transfer learning for ECG classification without the introduction of auxiliary domains as the bridge between the source and target domains. Related works of distant transfer learning in other areas were studied, which provided insights for our design and formulation for the proposed distant transfer learning algorithm.

#### 1.1.1. Traditional Transfer Learning for ECG Classification

Various works considered a knowledge transfer from a source model with different domains compared with the target domain. Transfer learning was performed to transfer knowledge from a pretrained model using the ImageNet database to the MIT-BIH arrhythmia database [11]. Before model construction, the continuous wavelet transform was used to transform the 1D ECG signals to 2D ECG signals. The input signals were passed to a convolutional neural network (CNN) algorithm for feature extraction and classification. The algorithm achieved a sensitivity of 96.2%, a specificity of 99.3%, and an accuracy of 99.1%. The accuracy was enhanced by 0.507% to 1.86% compared with existing works. Another work [12] also applied transfer learning to the MIT-BIH arrhythmia database. Three pretrained models, AlexNet, ResNet18, and GoogleNet, were selected for analysis. These models facilitated the knowledge transfer to the target model based on the CNN. The CNN model was optimized using different optimizers such as RMSprop, Adam, and SGDM. A performance evaluation showed that the AlexNet-CNN achieved the best accuracy with RMSprop (98.5%), the ResNet18-CNN achieved the best accuracy with SGDM (99.5%), and the GoogleNet-CNN achieved the best accuracy with Adam (98.6%). To prepare 2D ECG signals, the short-time Fourier transform was employed [13]. EfficientNet was selected as the pretrained model to support CNN-based target model construction for the MIT-BIH arrhythmia database and the PTB Diagnostic ECG database. An ablation study showed that the average accuracy improved from 94.7% to 97.0%.

For existing works using traditional machine learning-based classifiers, a preliminary study with 294 ECG samples (a small portion of six benchmark datasets) was carried out for ECG classification [14]. The continuous wavelet transform was firstly applied to prepare 2D ECG signals. ResNet50 was served as a pretrained model to fine-tune the target model. Three algorithms, namely XGBoost, random forest, and Softmax, were chosen to build the classifiers. These classifiers yielded an accuracy of 98.3%, 94.9%, and 93.2%, respectively.

The abovementioned works considered source and target domains that were not similar. In [15], a pretrained model for right bundle branch block classification was transferred to Brugada syndrome classification. The CNN and bidirectional long short-term memory (Bi-LSTM) were two core components for the ECG classification problem. An ablation study showed that the sensitivity of the model could be improved from 79.2% to 87.6%, whereas the specificity kept constant at 69.6%. Another work [16] presented a pretrained model using various datasets from different hospitals. Knowledge was transferred to a hybrid CNN and autoencoder target model. The design of the model was able to suppress the noise level of ECG signals and, thus, improve the model accuracy from 94.5% to 98.9%.

#### 1.1.2. Distant Transfer Learning Applications

A distant-domain high-level feature fusion approach was proposed for distant transfer learning [17]. The source domain was from one of the benchmark datasets, namely Dslr, Webcam, Amazon, and Catech-256. The auxiliary domain was based on breast ultrasound images, and the target domain was based on thyroid ultrasound images. Ablation studies showed that distant transfer learning improved the accuracy of the target model from 82.5% to 86.7% (Dslr), 79.5% to 84.4% (Webcam), 76.1% to 83.4% (Amazon), and 78.5% to 82.7% (Catech). The work [18] adopted these four source datasets for multiple-dataset distant transfer learning using distant feature fusion and the reduced-size Unet Segmentation model. Chest X-rays were selected as the auxiliary dataset for the bridge between multiple-source domains and target domains (COVID-19 computed tomography). An ablation study concluded that there was an accuracy improvement of the model from 86% to 96%.

Differed from the common assumption of distant transfer learning that the source and target domains are different, the work [19] considered the adoption of distant transfer learning when both the source and target domains were similar (industrial fault samples). A transitive distant-domain adaptation network was proposed for the transitive exploration of distant-domain samples. The average accuracy was 81.4% for five types of faults. Another work [20] proposed an autonomous machine learning pipeline with a feature transfer. Three experiments were carried out for the performance evaluation of the algorithm in the research topics: (i) text classification, where the source domain (BERT dataset) transferred knowledge to the target domain (the toxic comment dataset or the spam-email classification dataset); (ii) image classification, where the source domain (the ImageNet dataset) transferred knowledge to the target domain (the Cifar-10 dataset or the CASIA-FaceV5 dataset); and (iii) audio classification, where the source domain (the Audioset dataset) transferred knowledge to the target domain (the ESC-50 dataset or the Speech Command dataset).

### 1.2. Limitations of the Existing Works

After the studies of the existing works in transfer learning for ECG classifications [11,12,13,14,15,16] and distant transfer learning applications [17,18,19,20], the major research limitations were illustrated as follows:

- There were inadequacies in the performance evaluation and analysis in some existing works that did not employ cross-validation [11,12,13,14,17,18,19] and did not conduct ablation studies on the components of the algorithms [11,12,14,19,20].
- There was a lack of research on distant transfer learning for ECG classifications.
- Some of the research works [19,20] in distant transfer learning that considered both the source and target domains were similar; however, traditional transfer learning algorithms with a lower model complexity can achieve a similar performance.
- The details of the design and the formulation of multiple-source datasets on the distant transfer learning process [18] were insufficient.
- There were limited discussions on negative transfer avoidance between source and target domains in the aspects of domain, instance, and feature.

### 1.3. Research Contributions of the Article

To address the research limitations, a generative-adversarial-network-based auxiliary domain with a domain-feature-classifier negative-transfer-avoidance (GANAD-DFCNTA) algorithm was proposed for the knowledge transfer from distant source to target domains. The research contributions of the article were summarized below. It is worth noting that k-fold cross-validation where k = 5 was adopted for the performance evaluation, and analysis and ablation studies were carried out to reveal the effectiveness of the components of the GANAD-DFCNTA algorithm.

- Distant transfer learning was newly applied for ECG classifications. Six benchmark ECG datasets were selected for the research studies.
- With the unrestricted discipline of the source domain in distant transfer learning, generative-adversarial-network-based auxiliary domains were designed using both the source and target datasets.
- To minimize the risk of negative transfer from the source model to the target model, a domain-feature-classifier negative-transfer-avoidance algorithm was proposed to minimize loss for domain reconstruction, feature extraction, and the classifier.
- The GANAD-DFCNTA algorithm improved the accuracy by 0.303–5.19% compared with existing works.
- An investigation was carried out on the extension of the GANAD-DFCNTA algorithm with multiple-source datasets. An evaluation showed that the target model can enhance the accuracy by 3.67 to 4.89% with multiple-source datasets.
- Ablation studies of the GANAD-DFCNTA algorithm revealed the improvement of the accuracy of the target model by 2.42–3.58%, 1.35–2.73%, 0.767–2.37%, and 1.72–2.90%, compared with DFCNTA, GANAD-FCNTA, GANAD-DCNTA, and GANAD-DFNTA algorithms, respectively.

### 1.4. Organization of the Article

The rest of the article was organized as follows: Section 2 presented the details of the design and formulation of the GANAD-DFCNTA algorithm. The summary of the benchmark datasets and the performance evaluation of the GANAD-DFCNTA algorithm can be found in Section 3. To evaluate the contributions of the components of the GANAD-DFCNTA algorithm, Section 4 shared the ablation studies that were carried out to remove the individual components in each study. This article was ended with a conclusion and future research directions in Section 5.

## 2. Methodology

The methodology begins with the overview of the proposed distant transfer learning. For the design and formulation of the GANAD-DFCNTA algorithm, the first part of the GANAD algorithm was firstly presented in Section 2.2, followed by the second part of the DFCNTA algorithm.

### 2.1. Overview of the Proposed Distant Transfer Learning Algorithm for ECG Classification

**Figure 1:**
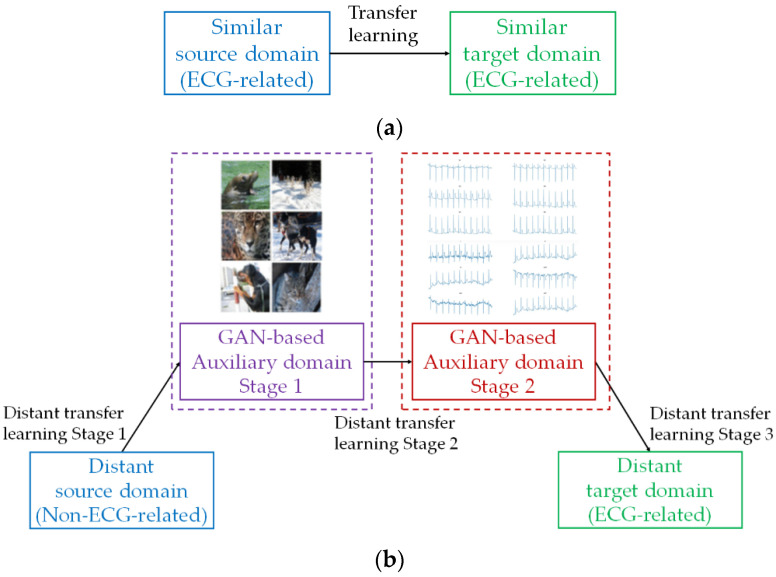
Conceptual architecture of transfer learning. (**a**) Traditional transfer learning, and (**b**) proposed distant transfer learning with auxiliary domains.

Figure 1 compares the conceptual architecture between traditional transfer learning for ECG classifications (Left) and the proposed distant transfer learning (Right). The former assumed that the source and target domains are similar, i.e., ECG-related datasets for both source and target datasets. The latter considered distant source and target domains. Since the desired application was ECG classification, the source domain was assumed as non-ECG-related, whereas the target domain was ECG-related. Exhaustive search and analysis (of infinitely many datasets) may be required for the selection of the appropriate auxiliary domain to serve as a bridge between the source and target domains. In our work, an algorithmic approach was selected to generate two auxiliary domains based on the employment of GAN by the source and target domains. One auxiliary domain was based on GAN with the source domain and another was based on GAN with the target domain. These auxiliary domains have the advantage of the generation of more relevant data and, thus, reduce the dissimilarities between the source domain and the target domain using the original formulation of direct distant transfer learning. As a result, the auxiliary domains contribute to negative transfer avoidance. In regard to the elements of positive transfer, attention was drawn into the nature of small-scale ECG datasets. The deep-learning-based ECG classifiers may experience overfitting and biased classification. Bringing large-scale image-based and text-based datasets for distant transfer learning could resolve the issues of overfitting and biased classification. The distant transfer learning process became a three-stage approach: (i) distant transfer learning stage 1, where knowledge was transferred from the distant source domain to the GAN-based auxiliary domain stage 1; (ii) distant transfer learning stage 2, where knowledge was transferred from the GAN-based auxiliary domain stage 1 to the GAN-based auxiliary domain stage 2; and (iii) distant transfer learning stage 3, where knowledge was transferred from the GAN-based auxiliary domain stage 2 to the target domain.

### 2.2. Generative-Adversarial-Network-Based Auxiliary Domains (GANAD) Algorithm

The generative adversarial network (GAN) has demonstrated its superiority in generating additional training data for the training of machine learning models [21,22,23]. One of the key advantages is the generation of additional training data for minority classes to reduce the impact of imbalanced classifications. To formulate the GAN for the two auxiliary domains (one relates to the source domain and one relates to the target domain), it is important to ensure good diversity for the generated data in order to enhance the functionality of the auxiliary domains as bridges between the source and target domains. Otherwise, without good diversity, the problem reduces to the traditional transfer learning process.

Starting with the formulation of the conditional GAN (cGAN) [24], there is a conditional mapping function named generator G:X→Y which uses the input x∈X to conditionally generate the output y∈Y. The latent variable z→Z is important to control the learning problem of multi-modal mapping G:X×Z→Y, such that an input x is mapped to diverse multiple outputs y. The minimization problem of the generator, with D as the discriminator, is given by:

(1) $$\underset{G}{\min}F_{G}\left(D,G\right)=E_{x,z}\left[\log\left(1-D\left(x,G\left(x,z\right)\right)\right)\right]$$

Equivalently, maximizing the score $D\left(x,G\left(x,z\right)\right)$ produces the outputs from the true data distribution. The discriminator aims at minimizing the score it gives to generated samples $G\left(x,z\right)$ with the minimization of the $D\left(x,G\left(x,z\right)\right)$ and the maximization of the $D\left(x,y\right)$ that it gives to the ground truth x. *G* attempts to fake *D* to believe that the generated samples are from *x*. This requires the formulation of the maximization problem of D as:

(2) $$\underset{D}{\max}F_{D}\left(D,G\right)=E_{x,y}\left[\log\left(D\left(x,y\right)\right)\right]+E_{x,z}\left[\log\left(1-D\left(x,G\left(x,z\right)\right)\right)\right]$$

Combining Equations (1) and (2), the loss function of the cGAN is defined as:

(3) $$\underset{G}{\min}\;\underset{D}{\max}F\left(D,G\right)=E_{x,y}\left[\log\left(D\left(x,y\right)\right)\right]+E_{x,z}\left[\log\left(1-D\left(x,G\left(x,z\right)\right)\right)\right]$$

In a typical formulation of GAN, mapping is learnt from a latent distribution *p_z_* to a complicated distribution. This requires a large-scale dataset. With the nature of small-scale ECG datasets in the target domain, increasing the network depth was not a feasible approach. Alternatively, enhancing the latent distribution for better modelling power was chosen. From [24], the diversity of the data can be enhanced by modifying the latent space with the Gaussian model:

(4) $$p_{z}\left(z\right)=\overset{N}{\underset{i=1}{\sum }}\omega_{i}f\left(z|\alpha_{i},\Sigma_{i}\right)$$

where $\omega =\left[\omega_{1},\ldots ,\omega_{N}\right]$ is the mixture weights vector and $f\left(z|\alpha_{i},\Sigma_{i}\right)$ is the probability of the sample *z* following normal distribution $N\left(\alpha_{i},\Sigma_{i}\right)$. If equal weights are assumed, Equation (4) is reduced to:

(5) $$p_{z}\left(z\right)=\overset{N}{\underset{i=1}{\sum }}\frac{f\left(z|\alpha_{i},\Sigma_{i}\right)}{N}$$

However, normal distribution has low kurtosis, and equal weights reduce the flexibility of mixture weights. It is also not practical to have equal importance for the generated samples because transferring knowledge between distant domains is more challenging with less relevant samples. In contrast to the traditional formulations of cGAN in Equations (1)–(5), in our work, we proposed the reformulation of Equation (4) with the logistic distribution $g\left(z|\mu ,\rho \right)$ with high kurtosis and the mixture weights vector $\beta =\left[\beta_{1},\ldots ,\beta_{N}\right]$ with unequal weights:

(6) $$p_{z}\left(z\right)=\overset{N}{\underset{i=1}{\sum }}\beta_{i}g\left(z|\mu ,\rho \right)$$

Individual samples can be obtained by:

(7) $$z=\gamma_{i}+\delta_{i}$$

where $\gamma_{i}$ is the deterministic function, $\delta_{i}$ is the covariance matrix, and σ is the auxiliary noise following logistic distribution. The model was then specified as:

(8) $$p_{data}\left(G\left(z\right)\right)=\overset{N}{\underset{i=1}{\sum }}\int \frac{p_{data}\left(G\left(\gamma_{i}+\delta_{i}\sigma \right)|\sigma \right)p\left(\sigma \right)}{N}d\sigma$$

with *N* logistic components.

For data generation, one of the *N* logistic components was selected and sample *z* was obtained from the selected component. The sample was passed to the generator to obtain the output. By applying L2 regularization to the generator, Equation (1) was updated to:

(9) $$\underset{G}{\min}F_{G}\left(D,G\right)=E_{x,z}\left[\log\left(1-D\left(x,G\left(x,z\right)\right)\right)\right]+\lambda \overset{N}{\underset{i}{\sum }}\beta_{i}{\left(1-\theta_{i}\right)}^{2}$$

where $\theta_{i}=\left[\theta_{i}{}_{1},\ldots ,\theta_{i}{}_{K}\right]$ is the K-dimensional covariance matrix for the selected ith logistic component and λ is the regularization hyperparameter.

Algorithm 1 summarizes the workflow for training *G* and *D* for GAN-based auxiliary domain stage 1 using the distant source domain. *G* generated a batch of synthetic non-ECG-related samples along with samples from the input non-ECG-related sample. The outmost for loop of Algorithm 1 iterated oversteps to train *G* and *D*. Every cycle completed the specific batch updates to *G* and *D*. The epoch was related to a cycle via the distant source dataset. The samples of the distant source dataset were utilized for the update of the weighting factor of the model in the minibatch.
**Algorithm 1**: GAN-based auxiliary domain stage 1**for** epoch *i = 1:L*
**do**  **for**
*D*, steps *j = 1:M*
**do**    Sample minibatch of size *p* from the ground truth sample of the distant source dataset    Sample minibatch of size *p* from the latent space    Applying gradient ascent to *D* to solve the maximization problem:    $\underset{D}{\max}F_{D}\left(D,G\right)=E_{x,y}\left[\log\left(D\left(x,y\right)\right)\right]+E_{x,z}\left[\log\left(1-D\left(x,G\left(x,z\right)\right)\right)\right]$  **end**
  **for**
*G*, steps *k = 1:N*
**do**    Sample minibatch of size *p* from the latent space    Applying gradient descent to *G* to solve the minimization problem:    $\underset{G}{\min}F_{G}\left(D,G\right)=E_{x,z}\left[\log\left(1-D\left(x,G\left(x,z\right)\right)\right)\right]+\lambda \overset{N}{\underset{i}{\sum }}\beta_{i}{\left(1-\theta_{i}\right)}^{2}$
  **end**
**end**

Likewise, Algorithm 2 summarizes the workflow to train *G* and *D* for GAN-based auxiliary domain stage 2 using the distant target domain. *G* generated a batch of synthetic ECG-related samples along with samples from the input ECG-related sample.
**Algorithm 2**: GAN-based auxiliary domain stage 2**for** epoch *i = 1:L*
**do**  **for**
*D*, steps *j = 1:M*
**do**    Sample minibatch of size *p* from the ground truth sample of the distant target dataset    Sample minibatch of size *p* from the latent space    Applying gradient ascent to *D* to solve the maximization problem:  $\underset{D}{\max}F_{D}\left(D,G\right)=E_{x,y}\left[\log\left(D\left(x,y\right)\right)\right]+E_{x,z}\left[\log\left(1-D\left(x,G\left(x,z\right)\right)\right)\right]$

  **end**
  **for**
*G*, steps *k = 1:N*
**do**    Sample minibatch of size *p* from the latent space    Applying gradient descent to *G* to solve the minimization problem:    
$\underset{G}{\min}F_{G}\left(D,G\right)=E_{x,z}\left[\log\left(1-D\left(x,G\left(x,z\right)\right)\right)\right]+\lambda \overset{N}{\underset{i}{\sum }}\beta_{i}{\left(1-\theta_{i}\right)}^{2}$

  **end**
**end**

### 2.3. Domain-Feature-Classifier Negative-Transfer-Avoidance (DFCNTA) Algorithm

In the distant transfer learning process (recall from Figure 1), there exists a feature space shared between the source and target domains in all stages. Referring to the basic formulations of the GAN loss $L_{GAN}$ and the classification loss $L_{class}\;$[25]:

(10) $$L_{GAN}\left(F,D\right)=E_{x_{u}\sim P_{T}\left(X\right)}\left[\log\left(D\left(F\left(x_{u}\right)\right)\right)\right]+E_{x_{s}\sim P_{S}\left(X\right)}\left[\log\left(1-D\left(F\left(x_{s}\right)\right)\right)\right]$$

(11) $$L_{class}\left(F,C\right)=E_{x_{l},y_{l}\sim T_{L}}\left[l_{class}\left(C\left(F\left(x_{l}\right)\right)\right),y_{l}\right]+E_{x_{s},y_{s}\sim S}\left[l_{class}\left(C\left(F\left(x_{s}\right)\right)\right),y_{s}\right]$$

where $x_{u}$ is the sample from the unlabeled target domain, $P_{T}\left(X\right)$ is the target marginal, *D* is the discriminator, $F\left(x_{u}\right)$ is the true feature, $x_{s}$ is the input sample from the labeled source domain, $P_{S}\left(X\right)$ is the source marginal, $F\left(x_{s}\right)$ is the fake feature, $x_{l}$ is the input sample from the labeled target domain based on the target joint $P_{T}\left(X,Y\right)$, $y_{l}$ is the output sample from the labeled target domain based on $P_{T}\left(X,Y\right)$, $T_{L}$ is the labeled target domain dataset, *C* is the classifier, $F\left(x_{l}\right)$ is the joint feature, $y_{s}$ is the output sample from the labeled source domain, and *S* is the labelled source set.

It is worth noting that the following assumption (Equation (12)) that every *x_s_* provides positive transfer to the target domain is not appropriate:

(12) $$P_{T}\left(Y|x_{t}\right)=P_{S}\left(Y|x_{s}\right)=P\left(Y|F\left(x_{t}\right)\right)=P\left(Y|F\left(x_{s}\right)\right)$$

Instead, there exists $x^{\prime }\epsilon X_{t}$. Equation (12) was revised as:

(13) $$P_{T}\left(Y|x_{t}\right)=P_{S}\left(Y|x_{s}\right)=P\left(Y|F\left(x_{t}\right)\right)=P\left(Y|F\left(x_{s}\right)\right)$$

To ensure good transferability of the marginal discriminator and the joint discriminator, a virtual label $y_{v}$ was defined so that a discriminator can served as the marginal discriminator and the joint discriminator. Particularly, the joint discriminator can use *x_u_* because the labeled data are limited. $y_{v}$ was passed to the feature network. Equation (10) was updated as:

(14) $$L_{GAN}\left(F,D\right)=E_{x_{u}\sim P_{T}\left(X\right)}\left[\log\left(D\left(F\left(x_{u}\right)\right)\right),y_{v}\right]+E_{x_{s}\sim P_{S}\left(X\right)}\left[\log\left(1-D\left(F\left(x_{s}\right)\right)\right),y_{v}\right]+E_{x_{l},y_{l}\sim T_{L}}\left[logD\left(F\left(x_{l}\right),y_{l}\right)\right]+E_{x_{s},y_{s}\sim S}\left[\log\left(1-D\left(F\left(x_{s}\right)\right),y_{s}\right)\right]$$

Since each sample in the source domain set may contribute to different extents to the positive transfer, weighting factors can be introduced to the second term of Equation (11). In [25,26,27], the weighting factors were estimated by the ratio between the target joint and the source joint. In our work, to improve diversity, the weighting factors φ were not restricted by this ratio. Instead, the factors were considered as hyperparameters to be optimized. Equation (11) was modified as:

(15) $$L_{class}\left(F,C\right)=E_{x_{l},y_{l}\sim T_{L}}\left[l_{class}\left(C\left(F\left(x_{l}\right)\right)\right),y_{l}\right]+\gamma E_{x_{s},y_{s}\sim S}\left[\varphi l_{class}\left(C\left(F\left(x_{s}\right)\right)\right),y_{s}\right]$$

where γ is the scaling factor to control φ.

As a result, the transfer learning problem became:

(16) $$\underset{F,C}{\mathrm{argmin}}\;\underset{D}{\mathrm{argmax}}L_{class}\left(F,C\right)-\sigma L_{GAN}\left(F,D\right)$$

where σ is the hyperparameter.

## 3. Benchmark Datasets and Performance Evaluation

This section first starts with a brief summary of the eight benchmark datasets in three data types: image, text, and ECG signal. The performance evaluation and the analysis of the GANAD-DFCNTA algorithm were shared. It was then compared with existing works.

### 3.1. Benchmark Dataset

Retrieving benchmark datasets is important for the performance evaluation of the GANAD-DFCNTA algorithm. For the source datasets, image-based and text-based datasets were selected because of their popularity and their successful previous works in transfer learning. More importantly, these datasets are not related to ECG classification and time-series data. For image-based datasets, ImageNet [28] and COCO [29] were chosen. For text-based datasets, WordNet [30] and Sentiment140 [31] were used. In regard to the target datasets, four ECG-based datasets were chosen, namely PTB-XL [32], the MIT-BIH arrhythmia database [33], the European ST-T database [34], and the long-term ST database [35]. Table 1 summarizes the details of the eight benchmark datasets. With the formulation of the GANAD-DFCNTA algorithm using multiple-source datasets (from 1 to 4) with ordering of datasets, 64 scenarios can be generated for each target dataset. Overall, 64 × 4 = 256 models were analyzed. The ECG signals performed ECG beat segmentation to obtain individual samples. Since the ECG beat segmentation is a well-known technique, we only highlighted the major steps where the full details can be referred to [36,37].

The general idea of the ECG beat segmentation is to locate all R waves so that individual ECG samples is defined as the small segment between two consecutive R waves. Given that the typical frequency range of QRS complexes is from 10 to 30 Hz. A high pass filter with transfer function *H_high_(z)* is firstly applied to the ECG signal:

(17) $$H_{high}\left(z\right)=\frac{1-2z^{-6}-64z^{-7}+z^{-12}+32z^{-16}+32z^{-18}}{32\left(1-2z^{-1}+z^{-2}\right)}$$

with delay and cutoff frequency of samples and 5 Hz, respectively.

A linear phase low-pass filter *H_low_(z)* is then applied to the ECG signal:

(18) $$H_{low}\left(z\right)=\frac{1-2z^{-6}+z^{-12}}{1-2z^{-1}+z^{-2}}$$

where the powerline noise and muscle noise can be significantly attenuated by 35 dB. The delay and the gain of the *H_low_(z)* are 5 samples and 31 dB, respectively.

To extract the slope information of the ECG signals (particularly QR and RS segments), a linear phase derivative filter with impulse response *h_d_[n]* is defined:

(19) $$h_{d}\left[n\right]=\left[-1,-2,2,1\right]$$

where the delay and gain are 2 samples and 14 dB, respectively.

It is followed by the employment of signal squaring (taking square) on the output. Afterwards, moving integration with difference equation *y_MI_[n]* is applied:

(20) $$y_{MI}\left[n\right]=\frac{\left[x\left[nT-\left(N-1\right)T\right]+x\left[nT-\left(N-2\right)T\right]+\cdots +x\left[nT\right]\right]}{N}$$

with the total number of samples N in the window.

The first sample of the output is the location of Q wave and the length of the output is the summation of twice QS segments and window width. Defining two thresholds, $\delta_{1}=P_{N}+0.25\left(P_{S}-P_{N}\right)$ and $\delta_{2}=0.5\delta_{1}$ with noise peak $P_{N}$ and signal peak $P_{S}$. The locations of Q waves, R waves, and S waves can be obtained.

Table 2 summarizes the sample size of each class for four ECG benchmark datasets [32,33,34,35].

### 3.2. Performance Evaluation and Analysis of the GANAD-DFCNTA Algorithm

#### 3.2.1. Two Auxiliary Domains

The ECG classification model was supported by the GANAD-DFCNTA and CNN algorithms. The formulations were based on interpatient ECG classification to align with the settings in existing works [11,12,13,14,15,16] in an apple-to-apple comparison. The architecture of the CNN is summarized as follows: layer 1—conv1D with kernel = 50, unit = 128, ReLU = 3, and strides = 3; layer 2—batch normalization; layer 3—maximum pooling with size = 2 and stride = 3; layer 4—conv1-D with kernel = 8, unit = 32, ReLU = 1, and strides = 1; layer 5—batch normalization; layer 6—maximum pooling with size = 2 and stride = 2; layer 7—conv1-D with kernel = 5, unit = 512, ReLU = 1, and strides = 1; layer 8—conv1-D with kernel = 3, unit = 128, ReLU = 1, and strides = 1; layer 9—fully connected layer; and layer 10—output layer. For all experiments, k-fold cross-validation with k = 5 was selected as a common order in classification problems [38,39,40]. Table 3 summarizes the performance of the best model for the multiple datasets (from 1 to 4) using specificity, sensitivity, and accuracy. These evaluation metrics are defined as follows:

(21) $$Specificity=\frac{TN}{N}$$

(22) $$Sensitivity=\frac{TP}{P}$$

(23) $$Accuracy=\frac{TP+TN}{P+N}$$

where TN is the true negative, TP is the true positive, N is the number of real negatives, and P is the number of real positives.

Since four distant source domains (two image-based [28,29] and two text-based domains [30,31]) were selected to perform distant transfer learning to enhance the performance of the target model for ECG classification problems, four models were built for [32,33,34,35]. An evaluation and an analysis were also conducted on the number of distant source domains and on the enhancement of the target domain. Therefore, four scenarios were set up with a different number of source domains using one dataset, two datasets, three datasets, and four datasets. In view of presenting the long list of the 25 pairs of sensitivity and specificity (25 classes in Table 2), Table 3, Table 4 and Table 5 summarize the overall sensitivity and specificity.

**Table 3:** Performance evaluation of the GANAD-DFCNTA algorithm.

	Specificity/Sensitivity/Accuracy (%)				
Target Dataset	Baseline	One Dataset	Two Datasets	Three Datasets	Four Datasets
PTB-XL [32]	92.8/93.7/93.3	93.8/94.5/94.2 with COCO	94.6/95.5/95.1 with COCO and ImageNet	95.7/96.4/96.1 with COCO, ImageNet, and Sentiment140	96.6/97.2/96.9
MIT-BIH Arrhythmia Database [33]	94.4/95.1/94.8	96.3/96.8/96.6 with COCO	97.5/98.1/97.8 with COCO and ImageNet	98.4/99.1/98.8 with COCO, ImageNet, and Sentiment140	99.1/99.7/99.4
European ST-T Database [34]	92.6/93.5/93.0	93.7/94.7/94.2 with ImageNet	94.8/95.5/95.2 with COCO and ImageNet	95.6/96.4/96.0 with COCO, ImageNet, and Sentiment140	96.4/97.1/96.8
Long-Term ST Database [35]	94.4/93.6/94.1	95.5/94.5/95.1 with COCO	96.3/95.4/95.9 with COCO and ImageNet	97.1/96.3/96.8 with COCO, ImageNet, and WordNet	97.9/97.0/97.5

**Table 4:** Performance evaluation of the GANAD-DFCNTA algorithm with one GAN-based auxiliar domain based on the source domain.

	Specificity/Sensitivity/Accuracy (%)				
Target Dataset	Baseline	One Dataset	Two Datasets	Three Datasets	Four Datasets
PTB-XL [32]	91.4/92.2/91.9	92.0/92.5/92.3 with COCO	93.1/93.8/93.5 with COCO and ImageNet	94.5/95.1/94.9 with COCO, ImageNet, and Sentiment140	95.6/96.1/95.9
MIT-BIH Arrhythmia Database [33]	92.6/93.4/93.0	94.5/95.2/94.8 with ImageNet	95.8/96.5/96.1 with COCO and ImageNet	96.7/97.5/97.1 with COCO, ImageNet, and Sentiment140	97.5/98.4/97.9
European ST-T Database [34]	90.6/91.7/91.1	91.9/93.0/92.4 with ImageNet	93.5/94.1/93.8 with COCO and ImageNet	93.8/94.7/94.3 with COCO, ImageNet, and Sentiment140	94.8/95.4/95.1
Long-Term ST Database [35]	92.3/91.7/92.0	93.5/92.7/93.1 with COCO	94.7/93.8/94.3 with COCO and ImageNet	95.5/94.3/95.0 with COCO, ImageNet, and WordNet	96.3/95.2/95.8

**Table 5:** Performance evaluation of the GANAD-DFCNTA algorithm with one GAN-based auxiliar domain based on the target domain.

	Specificity/Sensitivity/Accuracy (%)				
Target Dataset	Baseline	One Dataset	Two Datasets	Three Datasets	Four Datasets
PTB-XL [32]	90.7/91.6/91.2	91.3/92.1/91.8 with COCO	92.4/93.0/92.7 with COCO and ImageNet	93.9/94.4/94.2 with COCO, ImageNet, and Sentiment140	94.5/95.2/94.9
MIT-BIH Arrhythmia Database [33]	92.0/92.7/92.3	93.6/94.4/94.0 with ImageNet	96.1/94.9/96.4 with COCO and ImageNet	96.2/97.0/96.5 with COCO, ImageNet, and Sentiment140	96.6/97.3/96.9
European ST-T Database [34]	89.9/91.1/90.5	91.1/92.3/91.6 with COCO	92.8/93.3/93.0 with COCO and ImageNet	93.1/93.7/93.4 with COCO, ImageNet, and Sentiment140	93.6/94.3/93.9
Long-Term ST Database [35]	91.8/91.3/91.5	92.1/92.5/92.3 with COCO	93.9/93.1/93.5 with COCO and ImageNet	93.3/94.2/93.7 with COCO, ImageNet, and WordNet	95.4/94.4/94.9

The key observations were drawn as follows:

- Distant transfer learning via the GANAD-DFCNTA algorithm improved the performance (specificity, sensitivity, and accuracy) of the baseline ECG classification model. With more source datasets, the performance of the model can further be enhanced. It is worth noting that the saturation of model performance may be reached at some point, depending on the similarities between the source and target datasets.
- The percentage improvement of the specificity, sensitivity, and accuracy in PTB-XL was: 1.08, 0.854, and 0.965% for one dataset; 1.94, 1.92, and 1.93% for two datasets; 3.13, 2.88, and 3.00% for three datasets; 4.09, 3.74, and 3.86% for four datasets; and 1.02, 0.935, and 0.965% on average.
- The percentage improvement of the specificity, sensitivity, and accuracy in the MIT-BIH arrhythmia database was: 2.01, 1.79, and 1.90% for one dataset; 3.28, 3.15, and 3.19% for two datasets; 4.24, 4.21, and 4.22% for three datasets; 4.98, 4.84, and 4.89% for four datasets; and 1.25, 1.21, and 1.22% on average.
- The percentage improvement of the specificity, sensitivity, and accuracy in the European ST-T database was: 1.19, 1.28, and 1.25% for one dataset; 2.38, 2.14, and 2.22% for two datasets; 3.24, 3.10, and 3.18% for three datasets; 4.10, 3.85, and 4.02% for four datasets; and 1.03, 0.963, and 1.00% on average.
- The percentage improvement of the specificity, sensitivity, and accuracy in the long-term ST database was: 1.17, 0.962, and 1.07% for one dataset; 2.01, 1.92, and 1.96% for two datasets; 2.86, 2.88, and 2.87% for three datasets; 3.71, 3.63, and 3.67% for four datasets; and 0.928, 0.908, and 0.918% on average.
- The deviations between overall specificity and sensitivity with a varying number of datasets were 0.763% in PTB-XL, 0.613% in the MIT-BIH arrhythmia database, 0.842% in the European ST-T database, and 0.940% in long-term ST database.
- To better investigate the individual classes of highly imbalanced datasets [33], the overall deviations of the top five classes of the highest imbalanced ratios were 1.81% in Class 14, 1.45% in Class 13, 1.27% in Class 12, 1.13% in Class 11, and 1.03% in Class 10.
- As a remark, the baseline CNN algorithm serves as a common architecture that was adopted in many existing works. The main theme is the distant transfer learning process between distant multiple-source domains and target domains.

#### 3.2.2. One Auxiliary Domain

To reveal the benefits of two auxiliar domains, one less domain was formulated. The two scenarios were (i) a GAN-based auxiliary domain based on the source domain; and (ii) a GAN-based auxiliary domain based on the target domain. Similar to the settings of Section 3.2.1 with two auxiliary domains, Table 4 and Table 5 summarize the performance of the best model for the multiple datasets (from 1 to 4) using specificity, sensitivity, and accuracy for scenario (i) and scenario (ii), respectively. In both scenarios, the performance of the target models was enhanced with the increase in the number of datasets. Compared with the proposed GANAD-DFCNTA algorithm with two auxiliary domains, the formulation with one GAN-based auxiliar domain based on the source domain is less accurate, and a GAN-based auxiliar domain based on the target domain is least accurate. This revealed that both auxiliar domains were important to bridge the gap between domains using distant transfer learning.

- Cross-validation: Only single-split training and testing datasets were defined in related works [11,12,13,14]. Our work and [16] adopted 5-fold cross-validation.
- Ablation study: An ablation study was omitted in some works [11,12,14]. Our work and [13,16] included an ablation study to analyze the effectiveness of the individual components of the algorithm where multiple techniques were incorporated.
- Sensitivity and specificity: The differences between sensitivity and specificity were 3.22% for [11] which suggests a slightly biased classification towards the majority class. The differences in our work were ranged from 0.621 to 0.928%. Other works [12,13,14,16] did not report the sensitivity and specificity.
- Accuracy: Our work outperformed the existing work [11,13,14,16] by 0.303–5.19%. Compared with [12], our work enhanced the accuracy by 0.303–2.47% in 8 out of 9 scenarios.

### 3.3. Performance Comparison between GANAD-DFCNTA Algorithm and Existing Works

Table 6 compares the performance between GANAD-DFCNTA algorithm and existing works. For fair comparison, only target domains that were matched with the four benchmark datasets in [32,33,34,35] were included.

The discussion of the comparison was presented based on each item:

- Method: The basic architecture for ECG classification was typically the CNN, except [14] when using XGBoost. The CNN was a useful architecture that could automatically extract a feature and serve as a classifier.
- Source domain: In related works, the source domain was similar to the target domain in the field of ECG datasets. To the best of our knowledge, this work was the first work to consider distant transfer learning for ECG classifications with multiple distant source domains and target domains.
- Target domain: In related works, the MIT-BIH arrhythmia database [11,12,13,16] and the long-term ST database [14] were considered as the benchmark datasets in the target domain. Our work included two more benchmark datasets, the European ST-T database and the long–term ST database, for analysis.

To further analyze the effectiveness of the deep-learning-based algorithm, it was compared with traditional machine learning algorithms. Table 7 summarizes the results. It can be seen from the results that our work outperformed existing works with traditional machine learning in all the target ECG classification models. The accuracy of our GANAD-DFCNTA algorithm outperformed existing works by 36.3% in PTB-XL, 6.88% in the MIT-BIH arrhythmia database, 2.54–2.98% in the European ST-T database, and 2.96–8.94% in the long–term ST database. Therefore, it is worth formulating the ECG classification problem with deep learning, even though traditional machine learning has less model complexity.

**Table 6:** Performance comparison between GANAD-DFCNTA algorithm and existing deep-learning-based works.

Work	Method	Source Domain	Target Domain	Cross- Validation	Ablation Study	Specificity (%)	Sensitivity (%)	Accuracy (%)
[11]	Continuous wavelet transform and CNN	ImageNet	MIT-BIH Arrhythmia Database	No	No	96.2	99.3	99.1
[12]	RMSprop, Adam, SGDM optimizers, and CNN	ImageNet and COCO	MIT-BIH Arrhythmia Database	No	No	N/A	N/A	97.0 to 99.5
[13]	Short-time Fourier transform and CNN	ImageNet	MIT-BIH Arrhythmia Database	No	Yes	N/A	N/A	97.0
[14]	Continuous wavelet transform and XGBoost	COCO	MIT-BIH Arrhythmia Database and Long-Term ST Database	No	No	N/A	N/A	98.3
[16]	Autoencoder and CNN	Dataset from different hospitals	MIT-BIH Arrhythmia Database and	5-fold	Yes	N/A	N/A	94.5% to 98.9%
Our Work	GANAD-DFCNTA and CNN	ImageNet, COCO, WordNet, and Sentiment140	PTB-XL	5-fold	Yes	96.6	97.2	96.9
			MIT-BIH Arrhythmia Database,			99.1	99.7	99.4
			European ST-T Database, and			96.4	97.1	96.8
			Long-Term ST Database			97.9	97.0	97.5

## 4. Ablation Studies

Ablation studies of the GANAD-DFCNTA algorithm were carried out to evaluate the effectiveness of the components. Four algorithms, namely DFCNTA, GANAD-FCNTA, GANAD-DCNTA, and GANAD-DFNTA, were considered.

### 4.1. DFCNTA

To evaluate the effectiveness of the generative-adversarial-network-based auxiliary domains algorithm, we considered the DFCNTA algorithm for ECG classifications in the four target domains. Table 8 presents the performance of the DFCNTA algorithm.

The key observations were drawn as follows:

- The percentage improvement of the specificity, sensitivity, and accuracy in PTB-XL was: 0.323, 0.213, and 0.276% for one dataset; 0.647, 0.640, and 0.643% for two datasets; 1.08, 0.961, and 0.989% for three datasets; 1.29, 1.07, and 1.18% for four datasets; and 0.323, 0.268, and 0.295% on average.
- The percentage improvement of the specificity, sensitivity, and accuracy in the MIT-BIH arrhythmia database was: 0.424, 0.421, and 0.422% for one dataset; 0.742, 0.736, and 0.738% for two datasets; 0.953, 1.05, and 0.988% for three datasets; 1.27, 1.25, and 1.26% for four datasets; and 0.318, 0.313, and 0.315% on average.
- The percentage improvement of the specificity, sensitivity, and accuracy in the European ST-T database was: 0.324, 0.428, and 0.382% for one dataset; 0.756, 0.749, and 0.752% for two datasets; 0.972, 1.07, and 0.995% for three datasets; 1.30, 0.962, and 1.09% for four datasets; and 0.325, 0.241, and 0.273% on average.
- The percentage improvement of the specificity, sensitivity, and accuracy in the long-term ST database was: 0.424, 0.321, and 0.379% for one dataset; 0.742, 0.641, and 0.690% for two datasets; 0.953, 0.962, and 0.956% for three datasets; 1.27, 1.18, and 1.22% for four datasets; and 0.318, 0.295, and 0.305% on average.
- Consider four dataset-based scenarios: the GANAD-DFCNTA algorithm outperforms the DFCNTA algorithm by 2.77, 2.64, and 2.69% for PTB-XL; 3.66, 3.53, and 3.58% for the MIT-BIH arrhythmia database; 2.77, 2.53, and 2.71% for the European ST-T database; and 2.41, 2.43, and 2.42% for the long-term ST database.

**Table 8:** Performance evaluation of the DFCNTA algorithm.

	Specificity/Sensitivity/Accuracy (%)				
Target Dataset	Baseline	One Dataset	Two Datasets	Three Datasets	Four Datasets
PTB-XL [32]	92.8/93.7/93.3	93.1/93.9/93.5	93.4/94.3/93.9	93.8/94.6/94.2	94.0/94.7/94.4
MIT-BIH Arrhythmia Database [33]	94.4/95.1/94.8	94.8/95.5/95.2	95.1/95.8/95.5	95.3/96.1/95.7	95.6/96.3/96.0
European ST-T Database [34]	92.6/93.5/93.0	92.9/93.9/93.4	93.3/94.2/93.7	93.5/94.5/94.0	93.8/94.7/94.2
Long-Term ST Database [35]	94.4/93.6/94.1	94.8/93.9/94.4	95.1/94.2/94.7	95.3/94.5/94.9	95.6/94.7/95.2

To further investigate the effectiveness of the generative-adversarial-network-based auxiliary domains algorithm, the performance of Classes 10–14 in the MIT-BIH arrhythmia database [33] with and without the algorithm is summarized in Table 9. The key observations were explained as follows:

- The percentage improvement of the accuracy in Class 10 was: 29.9% for one dataset, 25.7% for two datasets, 25.6% for three datasets, 21.0% for four datasets, and 25.6% on average.
- The percentage improvement of the accuracy in Class 11 was: 28.5% for one dataset, 32.1% for two datasets, 31.6% for three datasets, 28.6% for four datasets, and 30.2% on average.
- The percentage improvement of the accuracy in Class 12 was: 40.3% for one dataset, 33.6% for two datasets, 34.7% for three datasets, 36.3% for four datasets, and 34.7% on average.
- The percentage improvement of the accuracy in Class 13 was: 44.8% for one dataset, 41.7% for two datasets, 43.8% for three datasets, 37.6% for four datasets, and 42.0% on average.
- The percentage improvement of the accuracy in Class 14 was: 153% for one dataset, 120% for two datasets, 56.8% for three datasets, 66.7% for four datasets, and 99.1% on average.

**Table 9:** Performance comparison between the GANAD-DFCNTA and DFCNTA algorithms.

	Accuracy (%)				
Algorithm	Class	One Dataset	Two Datasets	Three Datasets	Four Datasets
GANAD-DFCNTA	Class 10	74.7	78.2	83.9	87.1
	Class 11	70.3	75.7	81.6	84.0
	Class 12	68.9	73.1	78.8	82.3
	Class 13	62.7	68.3	74.5	77.9
	Class 14	47.5	55	58.8	62.5
DFCNTA	Class 10	57.5	62.2	66.8	72.0
	Class 11	54.7	57.3	62	65.3
	Class 12	49.1	54.7	58.5	60.4
	Class 13	43.3	48.2	51.8	56.6
	Class 14	18.8	25	37.5	37.5

The improvement was due to the contribution of the generation of additional training data to enhance the model. Particularly, the enhancement was more significant in the minority classes (for example, Classes 10–14 in Table 9). This aligned with the nature of machine learning problems where biased classification is usually towards the majority classes.

### 4.2. GANAD-FCNTA

To evaluate the effectiveness of the domain level in the domain-feature-classifier negative-transfer-avoidance algorithm, we considered the generative-adversarial-network-based auxiliary domains with the domain-feature-classifier negative-transfer-avoidance (GANAD-FCNTA) algorithm for ECG classifications in the four target domains. Table 10 presents its performance.

The key observations were drawn as follows:

- The percentage improvement of the specificity, sensitivity, and accuracy in PTB-XL was: 0.539, 0.467, and 0.502% for one dataset; 0.970, 0.961, and 0.965% for two datasets; 1.40, 1.28, and 1.34% for three datasets; 1.51, 1.49, and 1.50% for four datasets; and 0.378, 0.373, and 0.375% on average.
- The percentage improvement of the specificity, sensitivity, and accuracy in the MIT-BIH arrhythmia database was: 0.742, 0.736, and 0.739% for one dataset; 1.17, 1.16, and 1.16% for two datasets; 1.59, 1.79, and 1.69% for three datasets; 2.12, 2.10, and 2.11% for four datasets; and 0.53, 0.525, and 0.528% on average.
- The percentage improvement of the specificity, sensitivity, and accuracy in the European ST-T database was: 0.540, 0.642, and 0.610% for one dataset; 1.19, 1.18, and 1.18% for two datasets; 1.84, 1.93, and 1.89% for three datasets; 2.59, 2.57, and 2.58% for four datasets; and 0.648, 0.643, and 0.645% on average.
- The percentage improvement of the specificity, sensitivity, and accuracy in the long-term ST database was: 0.742, 0.641, and 0.689% for one dataset; 1.17, 1.07, and 1.11% for two datasets; 1.69, 1.71, and 1.70% for three datasets; 2.33, 2.24, and 2.27% for four datasets; and 0.583, 0.56, and 0.568% on average.
- Consider four dataset-based scenarios: the GANAD-DFCNTA algorithm outperformed the GANAD-FCNTA algorithm by 2.55, 2.21, and 2.32% for PTB-XL; 2.80, 2.68, and 2.73% for the MIT-BIH arrhythmia database; 1.47, 1.25, and 1.36% for the European ST-T database; and 1.35, 1.36, and 1.35% for the long-term ST database.

The findings reveal that the domain level-based negative transfer avoidance algorithm is important to the enhancement of the accuracy of the target model. Particularly, the dissimilar between the source and target domains in distant transfer learning is high, that requires the incorporation of domain-level information in the algorithm.

### 4.3. GANAD-DCNTA

To evaluate the effectiveness of the feature level in the domain-feature-classifier negative-transfer-avoidance algorithm, we considered the generative-adversarial-network-based auxiliary domains with the domain-feature-classifier negative-transfer-avoidance (GANAD-DCNTA) algorithm for ECG classifications in the four target domains. Table 11 presents its performance.

The key observations were drawn as follows:

- The percentage improvement of the specificity, sensitivity, and accuracy in PTB-XL was: 0.754, 0.534, and 0.643% for one dataset; 1.40, 1.39, and 1.39% for two datasets; 1.94, 1.81, and 1.86% for three datasets; 2.48, 2.13, and 2.25% for four datasets; and 0.62, 0.533, and 0.563% on average.
- The percentage improvement of the specificity, sensitivity, and accuracy in the MIT-BIH arrhythmia database was: 0.847, 0.841, and 0.844% for one dataset; 1.48, 1.37, and 1.42% for two datasets; 2.12, 2.21, and 2.17% for three datasets; 2.54, 2.42, and 2.47% for four datasets; and 0.635, 0.605, and 0.618% on average.
- The percentage improvement of the specificity, sensitivity, and accuracy in the European ST-T database was: 0.756, 0.856, and 0.802% for one dataset; 1.62, 1.93, and 1.81% for two datasets; 2.27, 2.46, and 2.35% for three datasets; 2.81, 2.99, and 2.88% for four datasets; and 0.703, 0.748, and 0.72% on average.
- The percentage improvement of the specificity, sensitivity, and accuracy in the long-term ST database was: 0.953, 0.855, and 0.899% for one dataset; 1.69, 1.60, and 1.65% for two datasets; 2.33, 2.35, and 2.34% for three datasets; 2.86, 2.88, and 2.87% for four datasets; and 0.715, 0.72, and 0.718% on average.
- Consider four dataset-based scenarios: the GANAD-DFCNTA algorithm outperformed the GANAD-FCNTA algorithm by 1.58, 1.57, and 1.57% for PTB-XL; 2.38, 2.36, and 2.37% for the MIT-BIH arrhythmia database; 1.26, 0.831, and 1.11% for the European ST-T database; and 0.824, 0.727, and 0.767% for the long-term ST database.

The findings revealed that the feature-level-based negative-transfer-avoidance algorithm enhanced the accuracy of the target model. Transferring new features from the source domain and retaining representative features in the target domain were two essential factors for the feature construction process.

### 4.4. GANAD-DFNTA

To evaluate the effectiveness of the classifier level in the domain-feature-classifier negative-transfer-avoidance algorithm, we considered the generative-adversarial-network-based auxiliary domains with the domain-feature-classifier negative-transfer-avoidance (GANAD-DFNTA) algorithm for ECG classifications in the four target domains. Table 12 presents its performance.

The key observations were drawn as follows:

- The percentage improvement of the specificity, sensitivity, and accuracy in PTB-XL was: 0.431, 0.320, and 0.375% for one dataset; 0.862, 0.747, and 0.785% for two datasets; 1.19, 1.07, and 1.13% for three datasets; 1.51, 1.39, and 1.45% for four datasets; and 0.378, 0.348, and 0.363% on average.
- The percentage improvement of the specificity, sensitivity, and accuracy in the MIT-BIH arrhythmia database was: 0.636, 0.631, and 0.633% for one dataset; 1.06, 1.05, and 1.05% for two datasets; 1.48, 1.68, and 1.58% for three datasets; 1.80, 2.00, and 1.90% for four datasets; and 0.45, 0.5, and 0.475% on average.
- The percentage improvement of the specificity, sensitivity, and accuracy in the European ST-T database was: 0.432, 0.535, and 0.538% for one dataset; 0.972, 1.07, and 1.02% for two datasets; 1.51, 1.60, and 1.55% for three datasets; 1.94, 2.03, and 1.98% for four datasets; and 0.485, 0.508, and 0.495% on average.
- The percentage improvement of the specificity, sensitivity, and accuracy in the long-term ST database was: 0.530, 0.534, and 0.532% for one dataset; 1.06, 1.07, and 1.06% for two datasets; 1.48, 1.50, and 1.49% for three datasets; 1.91, 1.92, and 1.91% for four datasets; and 0.478, 0.48, and 0.479% on average.
- Consider four dataset-based scenarios: the GANAD-DFCNTA algorithm outperformed the GANAD-DFNTA algorithm by 2.55, 2.32, and 2.43% for PTB-XL; 3.12, 2.78, and 2.90% for the MIT-BIH arrhythmia database; 2.12, 1.78, and 2.00% for the European ST-T database; and 1.77, 1.68, and 1.72% for the long-term ST database.

The findings revealed that the classifier-level-based negative-transfer-avoidance algorithm enhanced the accuracy of the target model. The fine tuning of the hyperparameters of the classifiers in the target model was crucial to ensure a positive transfer.

## 5. Conclusions

Distant transfer learning has received attention in recent years because the constraints of the high similarities between the source and target domains are released. Owning the fact that distant transfer learning has not yet been studied in ECG classification problems, this paper conducted a research study on the applicability of distant transfer learning for ECG classifications. A generative-adversarial-network-based auxiliary domain with the domain-feature-classifier negative-transfer-avoidance algorithm was proposed. Four benchmark distant-domain datasets were selected as source datasets, and four benchmark ECG datasets were selected as target datasets. A performance evaluation of the proposed algorithm showed that the accuracy improvement was 3.67 to 4.89% using four source datasets. Compared with existing works using traditional transfer learning, our work enhanced the accuracy of the ECG classification by 0.303–5.19%. Ablation studies on the generative-adversarial-network-based auxiliary domains algorithm with the domain-feature-classifier negative-transfer-avoidance algorithm also confirmed the effectiveness of the components.

As the first work to study distant transfer learning in ECG classifications with auxiliary domains, several future research directions were discussed: (i) investing an algorithm for the selection of appropriate distant source domains; (ii) investing an algorithm for the minimization of the number of distant source domains; (iii) merging similar and distant source domains to further enhance the performance of the target model; (iv) studying other baseline classification models; (v) investigating the performance of ECG classification models using varying settings such as personalized, interpatient, and intrapatient ECG classifications; (vi) enhancing the images with an image enhancement algorithm [48]; and (vii) proposing new variants of the generative adversarial network for data generation [49].

## Figures and Tables

**Table 1:** Summary of the benchmark datasets.

Dataset	Domain	Data Type	Description
ImageNet [28]	Source	Image	It contains about 14.2 million annotated images which are divided into more than 21,000 categories.
COCO [29]	Source	Image	It comprises about 330,000 images (>200,000 labeled images). The images involve 250,000 people and 1.5 million objects.
WordNet [30]	Source	Text	It has more than 150,000 index words which are categorized into adverbs, adjectives, verbs, and nouns. Every word can be attached to multiple-synonym sets (representing a semantic concept).
Sentiment140 [31]	Source	Text	It has about 1.6 million annotated tweets with positive, neutral, and negative polarities.
PTB-XL [32]	Target	ECG	It recruits 18,885 patients for the data collection of 21,837 clinical 12-lead ECGs.
MIT-BIH Arrhythmia Database [33]	Target	ECG	It comprises 48 30-min 2-channel ECGs from 47 volunteers.
European ST-T Database [34]	Target	ECG	It has 90 annotated ECGs from 79 subjects.
Long-Term ST Database [35]	Target	ECG	It collects 86 long-term ECG recordings of at least 21 h from 80 participants.

**Table 2:** Summary of the benchmark ECG datasets after ECG beat segmentation.

Dataset	Class	Sample Size
PTB-XL [32]	Class 0: Normal	118962
	Class 1: Myocardial infarction	68410
	Class 2: ST/T Change	65463
	Class 3: Conduction disturbance	61259
	Class 4: Hypertrophy	33108
MIT-BIH Arrhythmia Database [33]	Class 0: Normal	75052
	Class 1: Left bundle branch block	8075
	Class 2: Right bundle branch block	7259
	Class 3: Premature ventricular contraction	7130
	Class 4: Paced beat	7028
	Class 5: Atrial premature contraction	2546
	Class 6: Fusion of paced and normal beat	982
	Class 7: Fusion of ventricular and normal beat	803
	Class 8: Ventricular flutter wave	472
	Class 9: Nodal escape beat	229
	Class 10: Non-conducted P-wave	193
	Class 11: Aberrated atrial premature beat	150
	Class 12: Ventricular escape beat	106
	Class 13: Nodal premature beat	83
	Class 14: Atrial escape beat	16
European ST-T Database [34]	Class 0: Tachycardia	780606
	Class 1: Normal	431376
	Class 2: Bradycardia	32760
Long-Term ST Database [35]	Class 0: Normal	8832788
	Class 1: Myocardial ischemia	727956

**Table 7:** Performance comparison between GANAD-DFCNTA algorithm and traditional machine learning works.

Work	Method	Dataset	Cross- Validation	Ablation Study	Specificity (%)	Sensitivity (%)	Accuracy (%)
[41]	Few-shot learning with random forest	PTB-XL	5-fold	No	67.8	68.4	N/A
[42]	K-nearest neighbor,	PTB-XL	No	No	N/A	N/A	71.1
Our Work	GANAD-DFCNTA, and CNN	PTB-XL	5-fold	Yes	96.6	97.2	96.9
[43]	Support vector machine with SMOTE	MIT-BIH Arrhythmia Database	5-fold	Yes	N/A	N/A	93
[44]	Decision tree	MIT-BIH Arrhythmia Database	10-fold	No	98.6	88.6	N/A
Our Work	GANAD-DFCNTA and CNN	MIT-BIH Arrhythmia Database	5-fold	Yes	99.1	99.7	99.4
[45]	Complex support vector machine	European ST-T Database	No	No	N/A	N/A	94
[46]	Subspace k-nearest neighbor	European ST-T Database	10-fold	No	N/A	N/A	94.4
Our Work	GANAD-DFCNTA and CNN	European ST-T Database	5-fold	Yes	96.4	97.1	96.8
[47]	Support vector machine	Long-Term ST Database	5-fold	No	N/A	N/A	94.7
[47]	Neural network	Long-Term ST Database	5-fold	No	N/A	N/A	89.5
Our Work	GANAD-DFCNTA and CNN	Long-Term ST Database	5-fold	Yes	97.9	97.0	97.5

**Table 10:** Performance evaluation of the GANAD-FCNTA algorithm.

	Specificity/Sensitivity/Accuracy (%)				
Target Dataset	Baseline	One Dataset	Two Datasets	Three Datasets	Four Datasets
PTB-XL [32]	92.8/93.7/93.3	93.3/94.1/93.7	93.7/94.6/94.2	94.1/94.9/94.5	94.2/95.1/94.7
MIT-BIH Arrhythmia Database [33]	94.4/95.1/94.8	95.1/95.8/95.5	95.5/96.2/95.9	95.9/96.8/96.4	96.4/97.1/96.8
European ST-T Database [34]	92.6/93.5/93.0	93.1/94.1/93.6	93.7/94.6/94.1	94.3/95.3/94.8	95.0/95.9/95.4
Long-Term ST Database [35]	94.4/93.6/94.1	95.1/94.2/94.7	95.5/94.6/95.1	96.0/95.2/95.7	96.6/95.7/96.2

**Table 11:** Performance evaluation of the GANAD-DCNTA algorithm.

	Specificity/Sensitivity/Accuracy (%)				
Target Dataset	Baseline	One Dataset	Two Datasets	Three Datasets	Four Datasets
PTB-XL [32]	92.8/93.7/93.3	92.8/93.7/93.3	93.5/94.2/93.9	94.1/95.0/94.6	94.6/95.4/95.0
MIT-BIH Arrhythmia Database [33]	94.4/95.1/94.8	94.4/95.1/94.8	95.2/95.9/95.6	95.8/96.4/96.1	96.4/97.2/96.8
European ST-T Database [34]	92.6/93.5/93.0	92.6/93.5/93.0	93.3/94.3/93.8	94.1/95.3/94.7	94.7/95.8/95.2
Long-Term ST Database [35]	94.4/93.6/94.1	94.4/93.6/94.1	95.3/94.4/94.9	96.0/95.1/95.7	96.6/95.8/96.3

**Table 12:** Performance evaluation of the GANAD-DFNTA algorithm.

	Specificity/Sensitivity/Accuracy (%)				
Target Dataset	Baseline	One Dataset	Two Datasets	Three Datasets	Four Datasets
PTB-XL [32]	92.8/93.7/93.3	93.2/94.0/93.6	93.6/94.4/94.0	93.9/94.7/94.4	94.2/95.0/94.7
MIT-BIH Arrhythmia Database [33]	94.4/95.1/94.8	95.0/95.7/95.4	95.4/96.1/95.8	95.8/96.7/96.3	96.1/97.0/96.6
European ST-T Database [34]	92.6/93.5/93.0	93.0/94.0/93.5	93.5/94.5/94.0	94.0/95.0/94.5	94.4/95.4/94.9
Long Term ST Database [35]	94.4/93.6/94.1	94.9/94.1/94.6	95.4/94.6/95.1	95.8/95.0/95.5	96.2/95.4/95.9

## Data Availability

Not applicable.
